# Predisposition and Environment

**Published:** 1901-02

**Authors:** G. Alden Mills

**Affiliations:** New York


### PREDISPOSITION AND ENVIRONMENT.

BY DR. G. ALDEN MILLS, NEW YORK.

THROUGHOUT all the civilized world science is busied upon something that will give immunity from disease to the human race, but the natural man will never see that day. Physical laws to produce a perfect organism must have absolute harmony. The natural man was so created, and put into surroundings that would sustain such a condition, but prohibition broke that harmonious state and prohibition will never reinstate it. Tell a human being that he shall not, and he surely will. Self-will has brought, alas! too many into bondage to human passion in some form that rarely if ever can be reversed by the individual man as man. Man as man has no power to reclaim or to forgive himself. Man, it is true, is the highest order of creation under natural law. The holy writ gives us a picture of its richness and beauty in the garden of Eden; and man was made monarch of all that he surveyed, and he was given the entire animal kingdom for his subjects. In his mentality lay the power to subject all creatures. Sickness nor death knew any place. Immunity from both was perfect. Provision for reproduction was made ample. No wonder that the Creator exclaimed that it was good. This condition was placed in the hand of man with but one condition. Man was created with a power or choice. It was in the testing of this choice that harmony of the physical law received its infraction, and discord brought disease and death into existence. From that moment a shadow was cast that has continued to lengthen, and which has hung like a funeral pall over the entire human race till now, with no power of itself to lift itself away. Where did this shadow first manifest itself? This is a trite question at this point, for it opens the subject of this paper.

Can any one doubt for a moment that had generation commenced previous to this act of disobedience that the products would have been perfect? Every human being has the ability to know right and wrong. Consciousness of wrong came with the first act of disobedience. This led to uneasiness and fear and mental unrest. It needs no argument here to show us what results will be produced by these conditions continued. Cain was the first product of this uneasy pair; he was a murderer. The picture since that time is too dark to follow. To say that it is dark is to speak mildly. Look which way we may, it portends an ultimatum of utter defeat and failure to reinstate physical harmony. Sickness and death have ever since this fall prevailed with terrible destruction and devastation. The seed of disease has been sown, and it has continued to bring forth its kind. That which is born of the flesh will continue as it is. We can only palliate the effects, never eradicate. Now that we have taken our position, we will argue that it cannot be disproved. Not a few will deny our position, yet these denying ones dare not put to a test the truth that permeates the entire holy writ from Genesis to Revelation. "He that believeth my words shall have a witness in himself" that my words are the truth. We are not aiming at a sermon or theology; we are putting this subject upon a basis that cannot be shaken.

Assuming now that predisposition is plainly put before our minds, it is certain that all our efforts for eradication can only be, as we have said, palliative. We will not attempt to say that much is not being done for physical improvement, and that these efforts do not lessen the percentage of sickness and mortality. Predisposition to death comes because of this disobedience to the divine command. The wages of this transgression (sin) is death. In the first condition of mankind there was no such term as death. We shall argue that by violation of the command the spirit of the man and soul and body were thrown ajar. It can easily be surmised that the transgression brought a spirit of malignity which brought into play a spirit of revenge, and being brooded over became an impressible factor in the process of generation, and this became a matured trait in the character (or disposition) of Cain.

Here we see vividly the first act of predisposition manifested in an overt act, which we term murder. From our line of reasoning it is not difficult at all to follow out to a conclusion that all of our physical difficulties have their origin along the same line. Now Cain lives on and generates a race after his kind, and also the same with the children of Cain, and we have only to follow on a little period to note the dire results when destruction came upon the entire race, save eight souls. The history of the world shows a continuation of disease in a variety of forms that are not pleasant even to review. Just here we emphasize that the holy writ records that this predisposition was carried even to the fourth generation in specific disorders. We emphasize that there lurks continually what our none-too-much-honored friend, the late Professor Garretson, termed the "dis," that only needs favorable circumstances for a development. Therefore we assert that it will always be a life, in the natural, of contention with us. Disease is of satanic origin. It is an evil induction, and it can be overcome only by good, which is but another term for God. We stand face to face with human limitations in whatever direction we delve for knowledge. There is a line that the edict declares, "Hitherto shalt thou come, but no further."

Peter Cooper coined his immense fortune by the manufacture of glue, but he could not give us an analysis of it. The juices of the flesh are a hidden mystery to man's mind. Our bodies are organized into a complexity of organs that are under the supervision of function. It is along this line that what we term constitutional disease or disorders have their conception. So much are we the creatures of circumstances, the temperaments of our beings are effectually influenced and acted upon. At this point we see that environment is indicated. Environment brings out what predisposition makes possible. In our natural life we cannot wholly control our circumstances nor what may accrue from them. We may be led to extremes, either of which may result unfavorably. Dr. J. Leon Williams, of London, England, has given us what may be taken as an axiomatic statement, which cannot be disproved,—namely, "Predisposition and environment only make it possible for dental caries to thrive." We can only reduce them to a minimum result; to check them absolutely, never. We do not know at any moment what may overtake us and bring conditions that will tend to lessen the normal energy of the nervous system. Right here we wish to introduce a term of immense value, and one that will be comprehended readily by the majority. The late Professor Garretson was, I am quite sure, the author of this term "resistibility." It is, I think, a good term for nerve. We resist according to the nerve we have. We recall a remark by another much-valued colaborer, Professor Jonathan Taft. He says, "In approaching a case of pyorrhœa we should look into the face of the patient and determine what we have to help or hinder." Why in the face? Any one who has diagnostic ability to a greater or less degree will detect the probable amount of resistibility, or help or hinderance. If we find a condition which Professor Garretson termed "tubercular," we have a condition that forces us to recognize the results of a predisposition. At this point many stumble over an inability to cope with such a phase of disorder on the lines of belief that disease of this type is of a local origin, and the treatment will be a proof that only local treatment will not meet the demands of the case. The more we can bring to bear intelligent diagnostic ability in all our dealings with the organic structures, where either surgical or mechanical practice is to be applied, the larger will be our success along the line of betterment. Not infrequently is the organic structure overburdened because of a lack of needed discernment and proper judgment through the exhaustive strain of needless services.

This article is closed with the declaration, often made when asked, "Can you cure pyorrhœa, so called, or Riggs's disease?" we cannot cure a disease.

